# Metabolomics for plant breeding: knowledge without adoption

**DOI:** 10.3389/fpls.2026.1832662

**Published:** 2026-05-20

**Authors:** Patricia Pacheco-Ruiz, Sara Postacchini, Luca Mazzoni, José G. Vallarino

**Affiliations:** 1 Instituto de Hortofruticultura Subtropical y Mediterránea “La Mayora”, Departamento de Biología Molecular y Bioquímica, Universidad de Málaga-Consejo Superior de Investigaciones Científicas (IHSM-UMA-CSIC), Málaga, Spain; 2 Department of Agricultural, Food and Environmental Sciences (D3A), Università Politecnica delle Marche, Ancona, Italy

**Keywords:** annotation bottleneck, genomic selection (GS), genotype by environment (G × E) interaction, metabolomics, plant breeding, untargeted metabolomics, operational adoption

## 1 Introduction

No major commercial breeding program in any fruit, vegetable, or cereal crop has, to our knowledge, incorporated metabolomic data as a formal selection criterion in its operational pipeline. Metabolomics is used in breeding contexts: for characterizing diversity panels, for validating genomic predictions retrospectively, and for generating publishable results within academic-industry collaborations. But use as characterization is not adoption as selection. A formal selection criterion must survive the operational constraints of a breeding cycle: reproducibility across environments and years, interpretability by breeders who are not mass spectrometrists, and cost-effectiveness at the scale of hundreds to thousands of genotypes per cycle. By these standards, the translation deficit is complete.

The paradox is that the science, judged on its own terms, has delivered. Sakurai catalogued over 350 papers linking metabolomics to crop improvement that have been published since the early 2000s (Sakurai, 2022). Colantonio et al. demonstrated that targeted metabolomic profiles of sugars, acids, and volatiles, combined with consumer panel ratings, could predict sensory preferences in tomato and blueberry using machine learning models; when directly compared with genomic selection in a tomato panel of 70 accessions, metabolomic selection was markedly superior for all flavor attributes evaluated (Colantonio et al., 2022). Multi-omics integration for flavor has been accomplished in strawberry (Fan et al., 2022), and decision-support tools such as BreedingValue now allow breeders to rank genotypes using metabolomic data without statistical expertise (Senger et al., 2022). The analytical and statistical infrastructure exists. The barriers to adoption are not primarily technological; they are structural and diagnosing them requires examining three mechanisms that the literature has largely treated in isolation.

This gap between knowledge production and operational adoption is not without precedent. Genomic selection itself required nearly a decade from theoretical demonstration to routine deployment in animal and then plant breeding. But the analogy is imprecise. Genomic selection succeeded because genotyping costs fell by orders of magnitude, because the genotype is stable across environments, and because marker-trait associations, once estimated, transfer across populations with manageable loss of accuracy. None of these enabling conditions has an obvious metabolomic equivalent. The metabolomics case is instructive precisely because the instruments, statistical frameworks, and proof-of-concept data exist. What is missing is not technology but the structural conditions that would make adoption rational for a commercial breeder.

## 2 The GxE instability of the metabolome

The most fundamental barrier is that metabolomic profiles are environmentally labile to a degree that genomic markers are not. An SNP is an SNP regardless of whether the plant was grown in Huelva-Spain or in Florida-USA. A metabolite feature at m/z 449.108, tentatively annotated as cyanidin-3-O-glucoside, can vary two- to five-fold between the same genotype grown in consecutive seasons at the same location. We have recently documented this instability in strawberry: across two seasons and multiple cultivars, the proportion of metabolomic variance attributable to genotype-by-environment interaction exceeded that attributable to genotype alone for the majority of phenolic compounds (Pacheco-Ruiz et al., 2026). This is not a minor technical inconvenience. A metabolomic selection index calibrated in one environment may rank genotypes differently in another, precisely the kind of instability that breeders have spent decades learning to manage with genomic tools and multi-environment trials. For a breeding program evaluating thousands of genotypes per cycle, this instability is not a problem to be solved *post hoc*; it must be accounted for in the design of the selection system itself.

The standard response is that GxE can be modeled. This is true, but modeling demands replicated, multi-environment metabolomic data that almost no breeding program has generated, because the cost per sample remains an order of magnitude higher than genotyping. An SNP chip costs tens of dollars per sample; a single untargeted LC-MS run, including extraction, measurement, and data processing, costs hundreds. At the scale of a commercial program genotyping thousands of individuals per cycle, this difference is not incremental; it is prohibitive. Until the cost ratio changes, or until targeted panels reduce the metabolomic measurement to a handful of validated, low-cost markers, the GxE problem is not merely statistical but economic.

## 3 The annotation bottleneck

The second barrier compounds the first and is more insidious because it masquerades as a solvable technical problem. In any untargeted metabolomics experiment, fewer than 30% of features in a typical plant LC-MS dataset can be assigned even a tentative structural identity using current spectral databases (Allwood et al., 2011). The remaining majority are statistically real, often biologically interesting, and operationally useless for a breeder who needs to know what is being selected for and why. Breeding is a decision-making process under accountability: a breeder who selects for a genomic marker can point to a gene and a predicted function; a breeder who selects for an unannotated feature cluster correlated with consumer liking scores has a statistical association and nothing more. When that association fails to replicate, as it inevitably will for some features given the GxE problem, there is no mechanistic anchor to distinguish signal from noise. The annotation bottleneck thus compounds the GxE problem: unstable features that cannot be identified cannot be triaged, leaving the breeder to select blindly.

## 4 The absence of operational frameworks

The third barrier is perhaps the least discussed and the most consequential. Even where metabolomic data are stable and annotated, there is no consensus on how metabolomics should be integrated into genomic selection pipelines. Should metabolomic profiles serve as training phenotypes for genomic prediction models? Should they constitute independent selection indices weighted alongside genomic estimated breeding values? Should they function as culling criteria, metabolomic thresholds below which genotypes are discarded regardless of genomic merit? Each architecture implies different experimental designs, different data requirements, and different decision points in the breeding cycle. The literature contains examples of each approach in isolation, but no comparative evaluation within a single program and no operational manual that a breeder could adopt. This absence reflects a disciplinary gap: metabolomics researchers and quantitative geneticists read different journals, attend different conferences, and operate on different assumptions about what constitutes a useful result. The integration problem is as much sociological as it is methodological.

A separate trajectory has, however, demonstrated that metabolomic data can contribute productively to breeding without serving as a direct selection criterion. Metabolite genome-wide association studies (mGWAS) and metabolite quantitative trait locus (mQTL) mapping use metabolomic profiles as discovery phenotypes to identify genetic loci controlling metabolic variation. Once mapped, these loci can be incorporated into marker-assisted or genomic selection programs through standard SNP-based pipelines, at the cost and stability levels at which breeders already operate. This is the architecture in which metabolomic information has most clearly been translated into breeding practice. Li et al. (2025), for instance, used mGWAS in a panel of 452 edible maize accessions to identify hub loci controlling flavonoid and lipid variation, integrated these into a genomic selection model, and recovered an elite inbred line with the predefined nutritional and flavor profile. The metabolite itself does not enter the selection decision; its variation is used to enrich the genomic toolkit, after which the metabolomic measurement plays no further operational role. The implication is instructive. The metabolomic value proposition has been operationally realizable when the measurement is performed once, on a discovery panel, and converted into transferable genetic markers. It has not been realizable when the measurement must be repeated on every selection candidate in every cycle. The distinction is not a minor one of experimental design; it tracks the cost and stability constraints that define which technologies a breeding program can sustain.

The BreedingValue tool (Senger et al., 2022) represents the closest approximation to an operational framework: it converts metabolomic profiles into ranked genotype lists using a transparent weighting system. But BreedingValue assumes that its input data are stable across environments and that the weighting criteria reflect validated consumer or agronomic priorities, assumptions the tool itself cannot guarantee.

## 5 Discussion

The barriers described above are compounded by a deficit in the evidence base itself. The single most compelling proof-of-concept, Colantonio et al (Colantonio et al., 2022), was conducted within one breeding program, and no comparable study has appeared in another crop in the four years since publication. More fundamentally, neither Colantonio et al. nor BreedingValue (Senger et al., 2022) was designed to answer the question that commercial breeding programs need to answer: does metabolomic selection improve genetic gain per unit cost over a complete breeding cycle? Until that question is addressed empirically, the case for adoption rests on extrapolating from proof-of-concept to operational reality.

Recommending that breeders “should adopt metabolomics” would be vacuous without specifying the conditions under which adoption becomes rational. The first three conditions are technical and, given sufficient investment, achievable. First, targeted metabolomic panels, analogous to SNP chips in genomics, that measure a validated, cost-effective set of compounds directly relevant to breeding targets; untargeted metabolomics is a discovery tool, targeted panels are a deployment tool, and the transition from one to the other requires systematic validation across environments, which is the investment the field has not yet made. Second, multi-environment metabolomic datasets at a breeding-relevant scale: the GxE problem cannot be resolved with better statistical models alone but requires data from multiple locations and years, collected on populations large enough to estimate variance components reliably. This is expensive, unglamorous, and publishable only in breeding journals, which may explain why it has not been prioritized. Third, explicit integration architectures that specify how metabolomic information enters the selection decision at defined points in the breeding cycle.

The fourth condition is not technical. It requires the field to confront a question it has avoided: for how many crops, and for how many breeding targets, does metabolomic information provide sufficient added value over genomic selection alone to justify its cost? The field has been sustained by the implicit assumption that more data is always better. In an operational breeding context, more data is better only if the marginal information gain exceeds the marginal cost, and cost here includes not only the per-sample expense of metabolomic measurement, but the expertise required to generate, process, and interpret the data, and the opportunity cost of resources diverted from other selection tools. For traits where genomic prediction is already accurate and cost-effective, the rational decision may be not to adopt metabolomics at all.

Two decades of metabolomics-for-breeding research have produced genuine scientific advances and an impressive publication record. They have not produced a single operational adoption. At some point, the absence of adoption ceases to be a problem of technology transfer and becomes evidence that the value proposition has not been demonstrated at the scale that matters. The number of publications advocating metabolomics for breeding continues to grow while the number of breeding programs implementing it remains at zero; the widening of this gap warrants more scrutiny than it has received. The field must decide whether metabolomics-for-breeding is a viable operational program or a program of publications. Both are legitimate, but they require different investments, different success criteria, and different levels of honesty about what has been achieved.

## References

[B1] Allwood J. W. De Vos R. C. H. Moing A. Deborde C. Erban A. Kopka J. . (2011). Plant metabolomics and its potential for systems biology research background concepts, technology, and methodology. Methods Enzymology 500, 299–336. doi: 10.1016/B978-0-12-385118-5.00016-5, PMID: 21943904

[B2] Colantonio V. Ferrão L. F. V. Tieman D. M. Bliznyuk N. Sims C. Klee H. J. . (2022). Metabolomic selection for enhanced fruit flavor. Proc. Natl. Acad. Sci. U.S.A. 119, e2115865119. doi: 10.1073/pnas.2115865119, PMID: 35131943 PMC8860002

[B3] Fan Z. Tieman D. M. Knapp S. J. Zerbe P. Famula R. Barbey C. R. . (2022). A multi-omics framework reveals strawberry flavor genes and their regulatory elements. New Phytol. 236, 1089–1107. doi: 10.1111/nph.18416, PMID: 35916073 PMC9805237

[B4] Li C. Li Z. Lu B. Shi Y. Xiao S. Dong H. . (2025). Large-scale metabolomic landscape of edible maize reveals convergent changes in metabolite differentiation and facilitates its breeding improvement. Mol. Plant 18, 619–638. doi: 10.1016/j.molp.2025.02.007, PMID: 40025737

[B5] Pacheco-Ruiz P. Vallarino J. G. Zuñiga-Mayo V. M. Mazzoni L. Mezzetti B. Osorio S. . (2026). Genotype-by-environment interaction shapes the phenolic and volatile profile of strawberry fruit across seasons. Food Chem. 507, 148161. doi: 10.1016/j.foodchem.2026.148161, PMID: 41633166

[B6] Sakurai N. (2022). Recent applications of metabolomics in plant breeding. Breed. Sci. 72, 56–65. doi: 10.1270/jsbbs.21065, PMID: 36045891 PMC8987846

[B7] Senger E. Osorio S. Olbricht K. Shaw P. Denoyes B. Davik J. . (2022). Towards smart and sustainable development of modern berry cultivars in Europe. Plant J. 111, 1238–1251. doi: 10.1111/tpj.15876, PMID: 35751152

